# Supplementary material for: A large modulation of electron-phonon coupling and an emergent superconducting dome in doped strong ferroelectrics
Source: Nat Commun. 2021 Apr 19;12:2314. doi: 10.1038/s41467-021-22541-1 (PMC8055897; doi:10.1038/s41467-021-22541-1)
Supplement: Supplementary file 1 — Supplementary Information [file 41467_2021_22541_MOESM1_ESM.pdf]

Supplementary Information: A large modulation of electron-phonon coupling and an emergent superconducting dome in doped strong ferroelectrics

Jiaji Ma[1], Ruihan Yang[1,2], and Hanghui Chen[1,3]

[1]*NYU-ECNU Institute of Physics, NYU Shanghai, Shanghai 200122, China*

[2]*Donald Bren School of Information and Computer Science, UC Irvine, Irvine, CA 92697, USA*

[3]*Department of Physics, New York University, New York, NY 10003, USA*

Supplementary Note 1. CRYSTAL STRUCTURE AND COMPARISON TO EXPERIMENT

In Supplementary Table 1 we compare the lattice constants and spontaneous polarization of undoped tetragonal $BaTiO_3$ calculated to the experimental data.

Supplementary Table 1. Lattice constants and spontaneous polarization of undoped tetragonal $BaTiO_3$ calculated in our work, compared to the experimental values.

	experimental values	our calculations
a (Å)	3.992 [1]	3.930
c/a	1.011 [1]	1.012
P (C/m^2)	0.26 [2]	0.26

Supplementary Note 2. ELECTRON-PHONON SPECTRAL FUNCTION AND ELECTRON-PHONON COUPLING

In this section, we provide a brief explanation of electron-phonon coupling calculations [3, 4]. For a given electronic structure $\{\epsilon_{n\mathbf{k}}\}$ and a phonon spectrum $\{\omega_{\mathbf{q}\nu}\}$, we can calculate the mode-resolved electron-phonon coupling $\lambda_{\mathbf{q}\nu}$ using the double-delta approximation:

$$\lambda_{\mathbf{q}\nu} = \frac{1}{\pi N_F} \frac{\mathrm{Im}\Pi_{\mathbf{q}\nu}}{\omega_{\mathbf{q}\nu}^2} \quad \text{and} \quad \mathrm{Im}\Pi_{\mathbf{q}\nu} = \pi\omega_{\mathbf{q}\nu} \sum_{ij} \int \frac{d\mathbf{k}}{V_{\mathrm{BZ}}} |g_{ij}^{\nu}(\mathbf{k},\mathbf{q})|^2 \delta(\epsilon_{j\mathbf{k}} - \epsilon_F)\delta(\epsilon_{i\mathbf{k}+\mathbf{q}} - \epsilon_F) \quad (1)$$

where N_F is the density of states at the Fermi level, $\mathrm{Im}\Pi_{\mathbf{q}\nu}$ is the imaginary part of the electron-phonon self-energy associated with a phonon mode $\mathbf{q}\nu$ ($\mathbf{q}$ is the phonon crystal momentum and ν is the phonon index). $\epsilon_{n\mathbf{k}}$ is the electronic band structure ($\mathbf{k}$ is the electron crystal momentum, n is the electronic band index), ϵ_F is the Fermi level, $\omega_{\mathbf{q}\nu}$ is the phonon frequency and V_{BZ} is the volume of electron Brillouin zone. $g_{ij}^{\nu}(\mathbf{k},\mathbf{q}) = \langle\psi_{i\mathbf{k}+\mathbf{q}}|\partial_{\mathbf{q}\nu}V|\psi_{j\mathbf{k}}\rangle$ are the electron-phonon matrix elements (i, j label different orbitals) where $\partial_{\mathbf{q}\nu}V$ is the derivative of the self-consistent potential associated with the phonon mode $\mathbf{q}\nu$ [5]. Combining the two equations in Eq. (1), we have:

$$\lambda_{\mathbf{q}\nu} = \frac{1}{N_F\omega_{\mathbf{q}\nu}} \sum_{ij} \int \frac{d\mathbf{k}}{V_{\mathrm{BZ}}} |g_{ij}^{\nu}(\mathbf{k},\mathbf{q})|^2 \delta(\epsilon_{j\mathbf{k}} - \epsilon_F)\delta(\epsilon_{i\mathbf{k}+\mathbf{q}} - \epsilon_F) \quad (2)$$

which explicitly shows that $\lambda_{\mathbf{q}\nu} \propto 1/\omega_{\mathbf{q}\nu}$.

With all the mode-resolved electron-phonon coupling $\{\lambda_{\mathbf{q}\nu}\}$ and a complete phonon spectrum $\{\omega_{\mathbf{q}\nu}\}$, we can define a total electron-phonon spectral function $\alpha^2F(\omega)$ as [6]:

$$\alpha^2F(\omega) = \frac{1}{2}\sum_{\nu}\int \frac{d\mathbf{q}}{\Omega_{\mathrm{BZ}}} \omega_{\mathbf{q}\nu}\lambda_{\mathbf{q}\nu}\delta(\omega - \omega_{\mathbf{q}\nu}) \quad (3)$$

where Ω_{BZ} is the volume of phonon Brillouin zone. $\alpha^2F(\omega)$ will be used in the Eliashberg equation and McMillan's formula.

The accumulative electron-phonon coupling $\lambda(\omega)$ is defined as:

$$\lambda(\omega) = 2\int_0^{\omega} \frac{\alpha^2F(\nu)}{\nu} d\nu \quad (4)$$

The total electron-phonon coupling λ is defined as:

$$\lambda = 2\int_0^{\infty} \frac{\alpha^2F(\nu)}{\nu} d\nu \quad (5)$$

Supplementary Note 3. MULTI-ORBITAL ELIASHBERG EQUATION AND MCMILLAN'S EQUATION

For a multi-orbital system, we define an orbital-resolved electron-phonon spectral function $\alpha^2 F_{ij}(\omega)$ as [3, 7]:

$$\alpha^2 F_{ij}(\omega) = \frac{1}{N_i(0)} \sum_{\mathbf{k},\mathbf{q},\nu} |g_{ij}^{\nu}(\mathbf{k},\mathbf{q})|^2 \delta(\epsilon_{j\mathbf{k}} - \epsilon_F)\delta(\epsilon_{i\mathbf{k}+\mathbf{q}} - \epsilon_F)\delta(\omega - \omega_{\mathbf{q}\nu}) \tag{6}$$

where $N_i(0)$ is the density of states of the i-th orbital at Fermi energy and $g_{ij}^{\nu}(\mathbf{k},\mathbf{q})$ are the electron-phonon matrix elements.

In our study, the Fermi surface of doped $BaTiO_3$ is small. Therefore we use a multi-orbital Eliashberg equation with isotropic approximation [3] to estimate the superconducting transition temperature:

$$Z_i(i\omega_n) = 1 + \frac{\pi T}{\omega_n} \sum_{m,j} \frac{\omega_m}{\sqrt{\omega_m^2 + \Delta_j^2(i\omega_m)}} \lambda_{ij}(n-m) \tag{7}$$

$$Z_i(i\omega_n)\Delta_i(i\omega_n) = \pi T \sum_{m,j} \frac{\Delta_j(i\omega_m)}{\sqrt{\omega_m^2 + \Delta_j^2(i\omega_m)}} [\lambda_{ij}(n-m) - \mu_{ij}^*] \tag{8}$$

where i, j label different orbitals, $Z_i(i\omega_n)$ and $\Delta_i(i\omega_n)$ are superconducting renormalization function and gap function for the i-th orbital, ω_n are the discrete Matsubara frequencies at temperature T, μ_{ij}^* is the Morel-Anderson pseudopotential, and $\lambda_{ij}(n-m)$ is defined as:

$$\lambda_{ij}(n-m) = 2\int_0^{\infty} d\omega \frac{\omega\alpha^2 F_{ij}(\omega)}{(\omega_n - \omega_m)^2 + \omega^2} \tag{9}$$

We see that the Eliashberg equations Eq. (7) and Eq. (8) admit the trivial solution $\Delta_i(i\omega_n) = 0$ at all temperatures. The highest temperature for which the Eliashberg equations admit nontrivial solutions $\Delta_i(i\omega_n) \neq 0$ defines the critical temperature T_c.

Besides directly solving the Eliashberg equation, a widely used method is to use the McMillan's formula to estimate the superconducting transition temperature [8]:

$$T_c = \frac{\omega_{\log}}{1.2} \exp\left(- \frac{1.04(1+\lambda)}{\lambda - \mu^*(1+0.62\lambda)} \right) \tag{10}$$

where λ is the total electron-phonon coupling, μ^* is the Morel-Anderson pseudopotential

which is treated as an empirical parameter and $\omega_{\log}$ is:

$$\omega_{\log} = \lim_{n\to 0}\langle\omega^n\rangle = \exp\langle\ln\Omega\rangle \tag{11}$$

$$\langle\ln\Omega\rangle = \frac{2}{\lambda}\int d\Omega\,\alpha^2 F(\Omega)\frac{\ln\Omega}{\Omega} \tag{12}$$

We also use the McMillian's formula to estimate the superconducting transition temperature for doped $BaTiO_3$ using $\mu^* = 0.1$. The results are shown in Supplementary Fig. 1. We note that because the McMillian's formula is a fitting based on the single-orbital Eliashberg equation, it predicts a higher superconducting transition temperature than the three-orbital Eliashberg equation when the same value of Morel-Anderson pseudopotential μ^* is used. But the McMillian's formula also finds a very similar superconducting "dome" as a function of concentration in doped $BaTiO_3$.

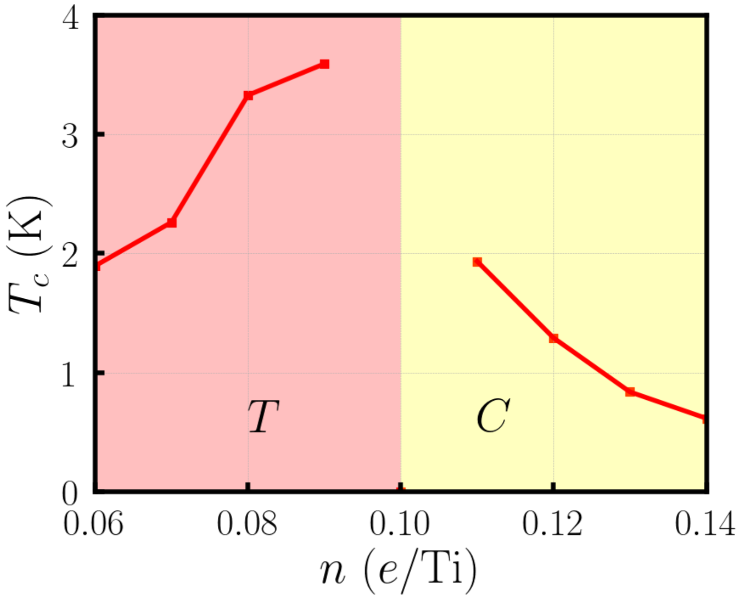

Supplementary Fig. 1. Superconducting transition temperature of doped $BaTiO_3$ as a function of electron concentration, calculated by the McMillan's formula using the Morel-Anderson pseudopotential $\mu^* = 0.1$.

Supplementary Note 4. CONVERGENCE TESTS

In this section, we test the convergence for **k**-mesh, **q**-mesh and Wannier function extrapolation. We use $0.11e$/f.u. as a representative electron doping concentration. Supplementary Fig. 2**a** shows the convergence of electronic density of states with respect to the **k**-mesh. Supplementary Fig. 2**b** shows the convergence of phonon density of states with respect to the **k**-mesh and **q**-mesh. We find that a **k**-mesh of $12 \times 12 \times 12$ and a **q**-mesh of $6 \times 6 \times 6$ are sufficient to converge the results. Based on this, we extrapolate the **k**-grid and **q**-grid using the maximally localized Wannier functions and calculate the electron-phonon spectral function $\alpha^2 F(\omega)$ and the total electron-phonon coupling λ using the fine **k**-mesh and **q**-mesh. We find that the electron-phonon spectral function $\alpha^2 F(\omega)$ and the total electron-phonon coupling λ are converged when both **k**-mesh and **q**-mesh are $50 \times 50 \times 50$.

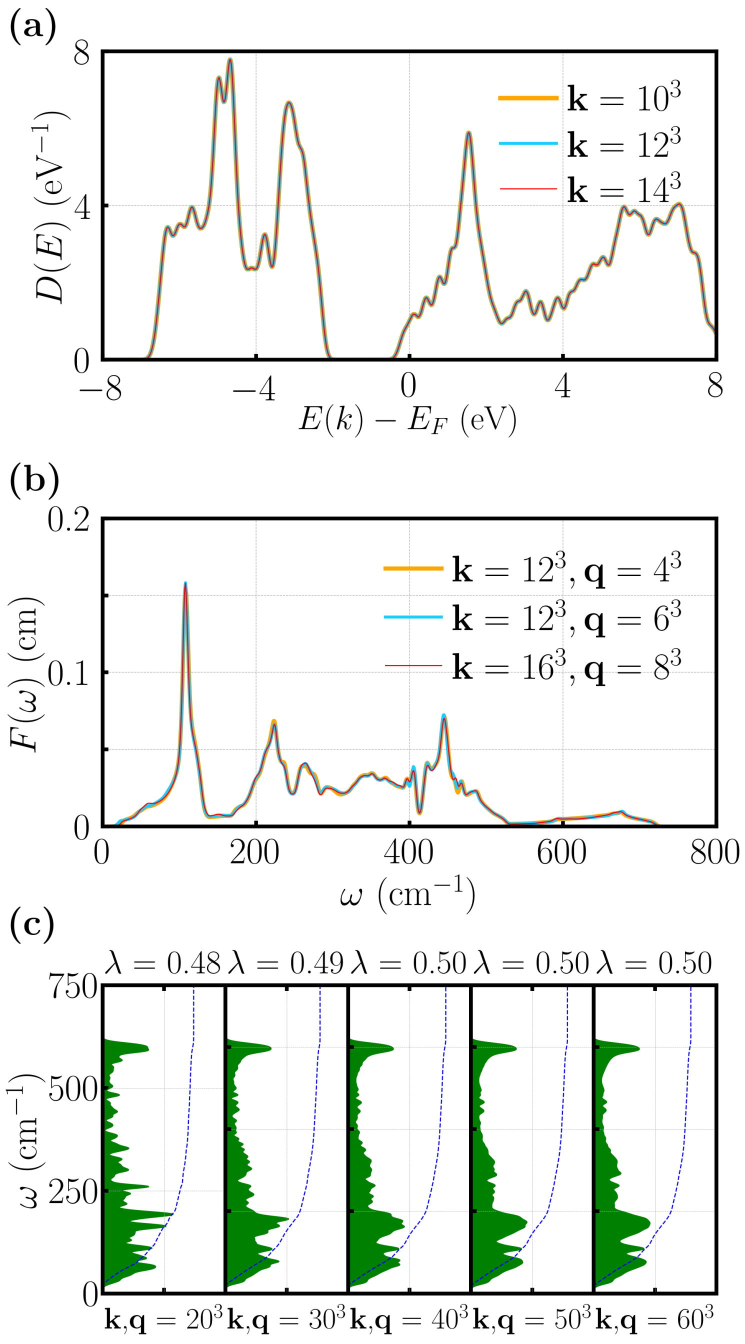

Supplementary Fig. 2. Test on doped $BaTiO_3$ at $0.11e$/f.u. concentration. **a**) The convergence of the electronic density of states with respect to the **k**-mesh. **b**) The convergence of the phonon density of states with respect to the **k**-mesh and **q**-mesh. **c**) The convergence of the electron-phonon spectral function $\alpha^2F(\omega)$ and the total electron-phonon coupling with respect to the Wannier extrapolated **k**-mesh and **q**-mesh. The coarse **k**-grid is $12 \times 12 \times 12$ and the coarse **q**-grid is $6 \times 6 \times 6$.

Supplementary Note 5. VALIDATION OF MIGDAL THEOREM

We estimate the Debye temperature T_D by using the full phonon spectrum to calculate the phonon heat capacity $C(T)$ per atom and then fitting the low-temperature $C(T)$ with $\frac{12\pi^4}{5}\left(\frac{T}{T_D}\right)^3 k_B$. On the other hand, because the Fermi surface is small, we estimate the Fermi temperature T_F by calculating the energy difference between the Fermi level and Ti conduction band edge.

In order for Migdal's theorem to be valid, the parameter $\lambda T_D/T_F$ needs to be much smaller than one [9]. Our calculations find that for doped $BaTiO_3$, $\lambda T_D/T_F$ is smaller than 0.1 in the range of concentrations from 0.06e/f.u. to 0.14e/f.u. This is a self-consistent check which indicates that within this range of concentrations, we can keep only the lowest order Feynman graph in evaluating the electron-phonon self-energy and ignore the vertex correction in calculating the electron-phonon coupling.

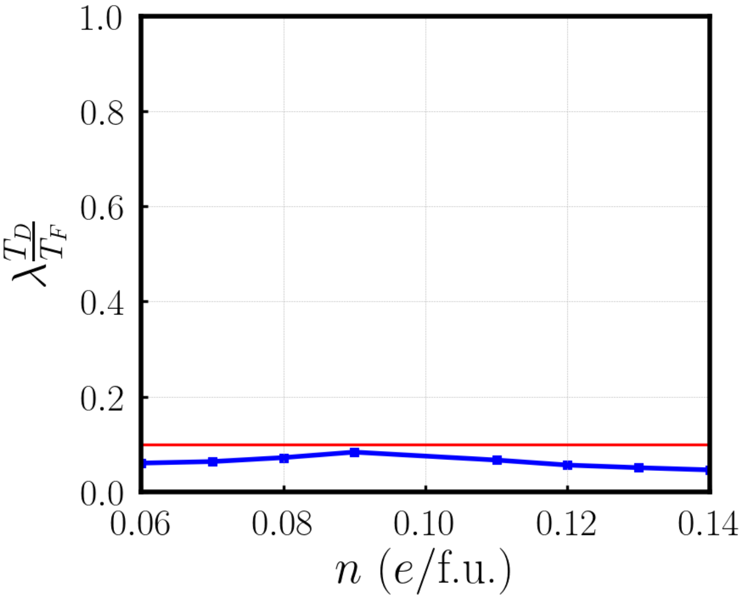

Supplementary Fig. 3. The parameter $\lambda T_D/T_F$ of doped $BaTiO_3$ at an electron concentration from 0.06e/f.u. to 0.14e/f.u. $\lambda T_D/T_F$ is smaller than 0.1 in this concentration range, indicating that the Migdal's theorem is valid.

Supplementary Note 6. MORE DETAILS ON THE STRUCTURAL AND PHONON PROPERTIES OF DOPED $BaTiO_3$ AROUND THE CRITICAL CONCENTRATION

In this section, we provide more details on the structural and phonon properties of doped $BaTiO_3$ close to the critical concentration.

When the electron doping concentration ranges from $0.06e$/f.u. and $0.14e$/f.u., we calculate the optimized tetragonal and cubic structures for doped $BaTiO_3$. The short and long axes (a and c) of the optimized tetragonal structure as well as the lattice constant a of the optimized cubic structure are shown in Supplementary Fig. 4**a**. We find that when the electron concentration is above the critical value $0.1e$/f.u., the tetragonal structure can not be stabilized and is reduced to the cubic structure. On the other hand, at an electron concentration below the critical value, while the cubic structure can be stabilized in the calculation by imposing symmetry, we find that it is physically unstable. Supplementary Fig. 4**b** shows the phonon spectrum of the optimized tetragonal and cubic structures. The cubic structure has imaginary phonon frequencies around Γ point, indicating its instability. The tetragonal structure does not have any imaginary phonon frequencies.

We note that electron-phonon coupling is only well-defined for a crystal structure that is physically stable (i.e. no imaginary phonon frequencies).

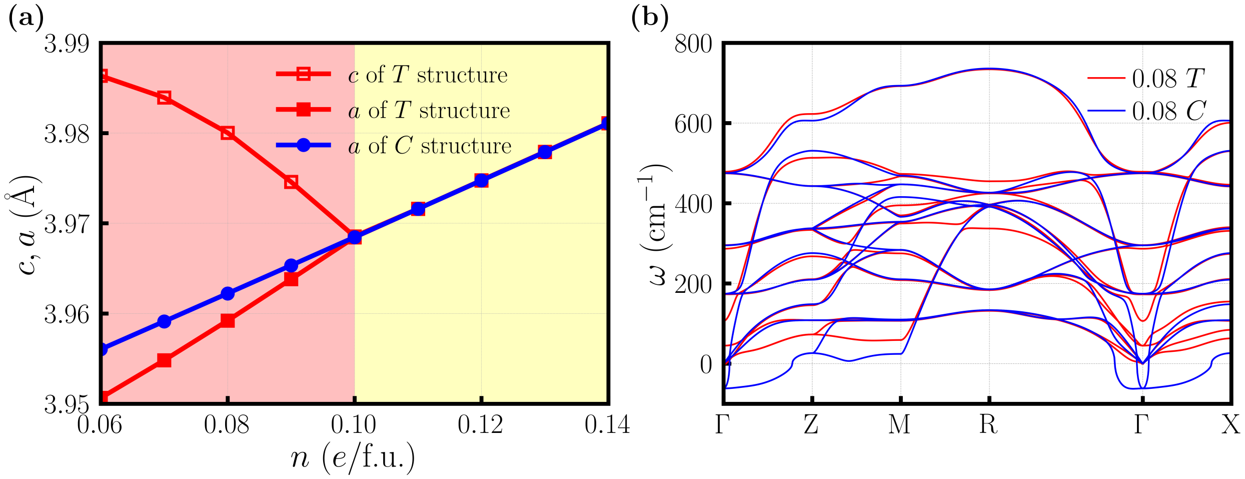

Supplementary Fig. 4. Doped $BaTiO_3$: **a**) the short and long axes (a and c) of the optimized tetragonal (T) structure as well as the lattice constant a of the optimized cubic (C) structure as a function of electron doping concentration, obtained from first-principles atomic relaxation calculations. The red open squares are the lattice constant c for the long axis of the T structure. The red solid squares are the lattice constant a for the short axis of the T structure. The blue circles are the lattice constant a of the C structure. **b**) The phonon dispersion of the tetragonal (T, red) and cubic (C, blue) structures at an electron concentration of $0.08e$/f.u. The optimized tetragonal structure is physically stable and has no imaginary phonon frequencies. The cubic structure, while stabilized in the calculation by imposing symmetry, is physically unstable hence having imaginary phonon frequencies around the Γ point.

Supplementary Note 7. STRUCTURAL PARAMETERS

In this section, we report the cell parameters and atom positions of all the stabilized crystal structures of doped $BaTiO_3$ at a given electron concentration.

We consider four possible crystal structures: rhombohedral (R), orthorhombic (O), tetragonal (T) and cubic (C). We find that when the carrier concentration n is less than $0.1e$/f.u., all the four crystal structures (R, O, T, C) can be stabilized during atomic relaxation by imposing symmetry. When the carrier concentration n is equal to or larger than $0.1e$/f.u., only the cubic structure (C) can be stabilized after atomic relaxation.

However, if the carrier concentration n is less than $0.025e$/f.u., the rhombohedral structure (R) has the lowest total energy. If the carrier concentration is larger than $0.025e$/f.u. but smaller than $0.1e$/f.u., the tetragonal structure (T) has the lowest total energy. Supplementary Fig. 5 clearly shows this phase boundary.

Therefore, we summarize the crystal structure information in four separate tables. Supplementary Table 2 shows the crystal structure information of the rhombohedral phase (R) when the electron concentration ranges from 0 to $0.09e$/f.u. Supplementary Table 4 shows the crystal structure information of the orthorhombic phase (O) when the electron concentration ranges from 0 to $0.09e$/f.u. Supplementary Table 5 shows the crystal structure information of the tetragonal phase (T) when the electron concentration ranges from 0 to $0.09e$/f.u. Supplementary Table 6 shows the crystal structure information of the cubic phase (C) when the electron concentration ranges from 0 to $0.14e$/f.u.

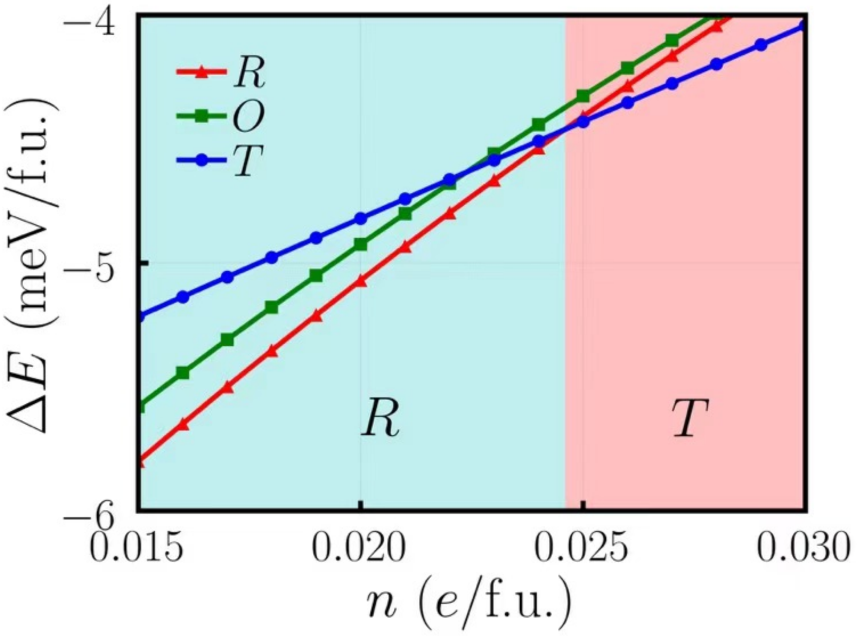

Supplementary Fig. 5. Phase diagram of doped $BaTiO_3$ as a function of electron concentration n. At each given electron concentration, the cubic structure C is used as the reference energy. The red triangles, green squares and blue circles correspond to rhombohedral (R), orthorhombic (O) and tetragonal (T) structures. The light blue (red) shade corresponds to the doping region in which the rhombohedral R (the tetragonal T) structure is the ground state.

Supplementary Table 2. The lattice parameters and ionic coordinates of doped $BaTiO_3$ in the rhombohedral structure (R). The electron concentration ranges from 0 to $0.09e$/f.u.

n (e/f.u.)	Phase	Cell Parameters	Atom Type	Wyckoff Site	X	Y	Z
0.00	R	$a = 3.948$Å<br>$b = 3.948$Å<br>$c = 3.948$Å<br>$\alpha = 89.934°$<br>$\beta = 89.934°$<br>$\gamma = 89.934°$	Ba1<br>Ti1<br>O1	3a<br>3a<br>9b	0.007<br>0.515<br>-0.007	0.007<br>0.515<br>0.498	0.007<br>0.515<br>0.498
0.01	R	$a = 3.950$Å<br>$b = 3.950$Å<br>$c = 3.950$Å<br>$\alpha = 89.937°$<br>$\beta = 89.937°$<br>$\gamma = 89.937°$	Ba1<br>Ti1<br>O1	3a<br>3a<br>9b	0.006<br>0.515<br>-0.007	0.006<br>0.515<br>0.498	0.006<br>0.515<br>0.498
0.02	R	$a = 3.952$Å<br>$b = 3.952$Å<br>$c = 3.952$Å<br>$\alpha = 89.942°$<br>$\beta = 89.942°$<br>$\gamma = 89.942°$	Ba1<br>Ti1<br>O1	3a<br>3a<br>9b	0.006<br>0.514<br>-0.006	0.006<br>0.514<br>0.498	0.006<br>0.514<br>0.498
0.03	R	$a = 3.954$Å<br>$b = 3.954$Å<br>$c = 3.954$Å<br>$\alpha = 89.948°$<br>$\beta = 89.948°$<br>$\gamma = 89.948°$	Ba1<br>Ti1<br>O1	3a<br>3a<br>9b	0.006<br>0.513<br>-0.006	0.006<br>0.513<br>0.498	0.006<br>0.513<br>0.498

0.04	R	$a = 3.956$Å<br>$b = 3.956$Å<br>$c = 3.956$Å<br>$\alpha = 89.954°$<br>$\beta = 89.954°$<br>$\gamma = 89.954°$	Ba1<br>Ti1<br>O1	3a<br>3a<br>9b	0.006<br>0.512<br>-0.005	0.006<br>0.512<br>0.499	0.006<br>0.512<br>0.499
0.05	R	$a = 3.959$Å<br>$b = 3.959$Å<br>$c = 3.959$Å<br>$\alpha = 89.961°$<br>$\beta = 89.961°$<br>$\gamma = 89.961°$	Ba1<br>Ti1<br>O1	3a<br>3a<br>9b	0.005<br>0.511<br>-0.005	0.005<br>0.511<br>0.499	0.005<br>0.511<br>0.499
0.06	R	$a = 3.961$Å<br>$b = 3.961$Å<br>$c = 3.961$Å<br>$\alpha = 89.968°$<br>$\beta = 89.968°$<br>$\gamma = 89.968°$	Ba1<br>Ti1<br>O1	3a<br>3a<br>9b	0.005<br>0.510<br>-0.004	0.005<br>0.510<br>0.499	0.005<br>0.510<br>0.499
0.07	R	$a = 3.963$Å<br>$b = 3.963$Å<br>$c = 3.963$Å<br>$\alpha = 89.977°$<br>$\beta = 89.977°$<br>$\gamma = 89.977°$	Ba1<br>Ti1<br>O1	3a<br>3a<br>9b	0.005<br>0.509<br>-0.003	0.005<br>0.509<br>0.500	0.005<br>0.509<br>0.500
0.08	R	$a = 3.964$Å<br>$b = 3.964$Å<br>$c = 3.964$Å<br>$\alpha = 89.986°$<br>$\beta = 89.986°$<br>$\gamma = 89.986°$	Ba1<br>Ti1<br>O1	3a<br>3a<br>9b	0.004<br>0.508<br>-0.002	0.004<br>0.508<br>0.500	0.004<br>0.508<br>0.500

0.09	R	$a = 3.967$Å<br>$b = 3.967$Å<br>$c = 3.967$Å<br>$\alpha = 89.992°$<br>$\beta = 89.992°$<br>$\gamma = 89.992°$	Ba1<br>Ti1<br>O1	3a<br>3a<br>9b	0.003<br>0.506<br>-0.001	0.003<br>0.506<br>0.501	0.003<br>0.506<br>0.501

Supplementary Table 4: The lattice parameters and ionic coordinates of doped $BaTiO_3$ in the orthorhombic structure (O). The electron concentration ranges from 0 to 0.09e/f.u. We note that because the cation-displacement is along [011] direction, the crystal structure of $BaTiO_3$ is base-centered orthorhombic (not simple orthorhombic).

n (e/f.u.)	Phase	Cell Parameters	Atom Type	Wyckoff Site	X	Y	Z
		$a = 3.927$Å					
		$b = 5.593$Å	Ba1	2a	0.000	0.007	0.007
		$c = 5.603$Å	Ti1	2b	0.500	0.518	0.518
0.00	O	$\alpha = 90°$	O1	2a	0.000	0.496	0.496
		$\beta = 90°$	O2	4e	0.500	0.497	-0.009
		$\gamma = 90°$					
		$a = 3.931$Å					
		$b = 5.594$Å	Ba1	2a	0.000	0.007	0.007
		$c = 5.604$Å	Ti1	2b	0.500	0.517	0.517
0.01	O	$\alpha = 90°$	O1	2a	0.000	0.497	0.497
		$\beta = 90°$	O2	4e	0.500	0.497	-0.008
		$\gamma = 90°$					
		$a = 3.935$Å					
		$b = 5.596$Å	Ba1	2a	0.000	0.007	0.007
		$c = 5.605$Å	Ti1	2b	0.500	0.516	0.516
0.02	O	$\alpha = 90°$	O1	2a	0.000	0.497	0.497
		$\beta = 90°$	O2	4e	0.500	0.498	-0.008
		$\gamma = 90°$					

0.03	O	$a = 3.940$Å<br>$b = 5.599$Å<br>$c = 5.607$Å<br>$\alpha = 90°$<br>$\beta = 90°$<br>$\gamma = 90°$	Ba1<br>Ti1<br>O1<br>O2	2a<br>2b<br>2a<br>4e	0.000<br>0.500<br>0.000<br>0.500	0.007<br>0.515<br>0.498<br>0.498	0.007<br>0.515<br>0.498<br>-0.007
0.04	O	$a = 3.944$Å<br>$b = 5.601$Å<br>$c = 5.608$Å<br>$\alpha = 90°$<br>$\beta = 90°$<br>$\gamma = 90°$	Ba1<br>Ti1<br>O1<br>O2	2a<br>2b<br>2a<br>4e	0.000<br>0.500<br>0.000<br>0.500	0.006<br>0.515<br>0.498<br>0.498	0.006<br>0.515<br>0.498<br>-0.007
0.05	O	$a = 3.948$Å<br>$b = 5.603$Å<br>$c = 5.609$Å<br>$\alpha = 90°$<br>$\beta = 90°$<br>$\gamma = 90°$	Ba1<br>Ti1<br>O1<br>O2	2a<br>2b<br>2a<br>4e	0.000<br>0.500<br>0.000<br>0.500	0.006<br>0.514<br>0.498<br>0.498	0.006<br>0.514<br>0.498<br>-0.006
0.06	O	$a = 3.952$Å<br>$b = 5.605$Å<br>$c = 5.610$Å<br>$\alpha = 90°$<br>$\beta = 90°$<br>$\gamma = 90°$	Ba1<br>Ti1<br>O1<br>O2	2a<br>2b<br>2a<br>4e	0.000<br>0.500<br>0.000<br>0.500	0.006<br>0.513<br>0.499<br>0.499	0.006<br>0.513<br>0.499<br>-0.006
0.07	O	$a = 3.956$Å<br>$b = 5.607$Å<br>$c = 5.611$Å<br>$\alpha = 90°$<br>$\beta = 90°$<br>$\gamma = 90°$	Ba1<br>Ti1<br>O1<br>O2	2a<br>2b<br>2a<br>4e	0.000<br>0.500<br>0.000<br>0.500	0.006<br>0.511<br>0.499<br>0.499	0.006<br>0.511<br>0.499<br>-0.005

0.08	O	$a = 3.960$Å					
		$b = 5.609$Å	Ba1	2a	0.000	0.005	0.005
		$c = 5.612$Å	Ti1	2b	0.500	0.510	0.510
		$\alpha = 90°$	O1	2a	0.000	0.500	0.500
		$\beta = 90°$	O2	4e	0.500	0.500	-0.004
		$\gamma = 90°$					
0.09	O	$a = 3.964$Å					
		$b = 5.611$Å	Ba1	2a	0.000	0.004	0.004
		$c = 5.612$Å	Ti1	2b	0.500	0.508	0.508
		$\alpha = 90°$	O1	2a	0.000	0.500	0.500
		$\beta = 90°$	O2	4e	0.500	0.500	-0.002
		$\gamma = 90°$					

Supplementary Table 5: The lattice parameters and ionic coordinates of doped $BaTiO_3$ in the tetragonal structure (T). The electron concentration ranges from 0 to $0.09e$/f.u.

n (e/f.u.)	Phase	Cell Parameters	Atom Type	Wyckoff Site	X	Y	Z
		$a = 3.930$Å					
		$b = 3.930$Å	Ba1	1a	0.000	0.000	0.015
0.00	T	$c = 3.977$Å	Ti1	1b	0.500	0.500	0.528
		$\alpha = 90°$	O1	2c	0.500	0.000	0.501
		$\beta = 90°$	O2	1b	0.500	0.500	0.994
		$\gamma = 90°$					
		$a = 3.933$Å					
		$b = 3.933$Å	Ba1	1a	0.000	0.000	0.015
0.01	T	$c = 3.981$Å	Ti1	1b	0.500	0.500	0.528
		$\alpha = 90°$	O1	2c	0.500	0.000	0.501
		$\beta = 90°$	O2	1b	0.500	0.500	0.994
		$\gamma = 90°$					
		$a = 3.936$Å					
		$b = 3.936$Å	Ba1	1a	0.000	0.000	0.015
0.02	T	$c = 3.984$Å	Ti1	1b	0.500	0.500	0.528
		$\alpha = 90°$	O1	2c	0.500	0.000	0.501
		$\beta = 90°$	O2	1b	0.500	0.500	0.994
		$\gamma = 90°$					
		$a = 3.939$Å					
		$b = 3.939$Å	Ba1	1a	0.000	0.000	0.015
0.03	T	$c = 3.986$Å	Ti1	1b	0.500	0.500	0.528
		$\alpha = 90°$	O1	2c	0.500	0.000	0.502
		$\beta = 90°$	O2	1b	0.500	0.500	0.994
		$\gamma = 90°$					

0.04	T	$a = 3.943$Å<br>$b = 3.943$Å<br>$c = 3.987$Å<br>$\alpha = 90°$<br>$\beta = 90°$<br>$\gamma = 90°$	Ba1<br>Ti1<br>O1<br>O2	1a<br>1b<br>2c<br>1b	0.000<br>0.500<br>0.500<br>0.500	0.000<br>0.500<br>0.000<br>0.500	0.015<br>0.527<br>0.502<br>0.994
0.05	T	$a = 3.947$Å<br>$b = 3.947$Å<br>$c = 3.988$Å<br>$\alpha = 90°$<br>$\beta = 90°$<br>$\gamma = 90°$	Ba1<br>Ti1<br>O1<br>O2	1a<br>1b<br>2c<br>1b	0.000<br>0.500<br>0.500<br>0.500	0.000<br>0.500<br>0.000<br>0.500	0.015<br>0.527<br>0.502<br>0.995
0.06	T	$a = 3.951$Å<br>$b = 3.951$Å<br>$c = 3.986$Å<br>$\alpha = 90°$<br>$\beta = 90°$<br>$\gamma = 90°$	Ba1<br>Ti1<br>O1<br>O2	1a<br>1b<br>2c<br>1b	0.000<br>0.500<br>0.500<br>0.500	0.000<br>0.500<br>0.000<br>0.500	0.014<br>0.525<br>0.503<br>0.995
0.07	T	$a = 3.955$Å<br>$b = 3.955$Å<br>$c = 3.984$Å<br>$\alpha = 90°$<br>$\beta = 90°$<br>$\gamma = 90°$	Ba1<br>Ti1<br>O1<br>O2	1a<br>1b<br>2c<br>1b	0.000<br>0.500<br>0.500<br>0.500	0.000<br>0.500<br>0.000<br>0.500	0.014<br>0.524<br>0.503<br>0.997
0.08	T	$a = 3.959$Å<br>$b = 3.959$Å<br>$c = 3.980$Å<br>$\alpha = 90°$<br>$\beta = 90°$<br>$\gamma = 90°$	Ba1<br>Ti1<br>O1<br>O2	1a<br>1b<br>2c<br>1b	0.000<br>0.500<br>0.500<br>0.500	0.000<br>0.500<br>0.000<br>0.500	0.013<br>0.521<br>0.504<br>0.998

0.09	T	$a = 3.964$Å<br>$b = 3.964$Å<br>$c = 3.975$Å<br>$\alpha = 90°$<br>$\beta = 90°$<br>$\gamma = 90°$	Ba1<br>Ti1<br>O1<br>O2	1a<br>1b<br>2c<br>1b	0.000<br>0.500<br>0.500<br>0.500	0.000<br>0.500<br>0.000<br>0.500	0.011<br>0.518<br>0.505<br>0.999

Supplementary Table 6: The lattice parameters and ionic coordinates of doped $BaTiO_3$ in the cubic structure (C). The electron concentration ranges from 0 to $0.14e$/f.u.

n (e/f.u.)	Phase	Cell Parameters	Atom Type	Wyckoff Site	X	Y	Z
0.00	C	$a = 3.938$Å<br>$b = 3.938$Å<br>$c = 3.938$Å<br>$\alpha = 90°$<br>$\beta = 90°$<br>$\gamma = 90°$	Ba1<br>Ti1<br>O1	1a<br>1b<br>3c	0.000<br>0.500<br>0.000	0.000<br>0.500<br>0.500	0.000<br>0.500<br>0.500
0.01	C	$a = 3.941$Å<br>$b = 3.941$Å<br>$c = 3.941$Å<br>$\alpha = 90°$<br>$\beta = 90°$<br>$\gamma = 90°$	Ba1<br>Ti1<br>O1	1a<br>1b<br>3c	0.000<br>0.500<br>0.000	0.000<br>0.500<br>0.500	0.000<br>0.500<br>0.500
0.02	C	$a = 3.944$Å<br>$b = 3.944$Å<br>$c = 3.944$Å<br>$\alpha = 90°$<br>$\beta = 90°$<br>$\gamma = 90°$	Ba1<br>Ti1<br>O1	1a<br>1b<br>3c	0.000<br>0.500<br>0.000	0.000<br>0.500<br>0.500	0.000<br>0.500<br>0.500
0.03	C	$a = 3.947$Å<br>$b = 3.947$Å<br>$c = 3.947$Å<br>$\alpha = 90°$<br>$\beta = 90°$<br>$\gamma = 90°$	Ba1<br>Ti1<br>O1	1a<br>1b<br>3c	0.000<br>0.500<br>0.000	0.000<br>0.500<br>0.500	0.000<br>0.500<br>0.500

0.04	C	$a = 3.950$Å<br>$b = 3.950$Å<br>$c = 3.950$Å<br>$\alpha = 90°$<br>$\beta = 90°$<br>$\gamma = 90°$	Ba1<br>Ti1<br>O1	1a<br>1b<br>3c	0.000<br>0.500<br>0.000	0.000<br>0.500<br>0.500	0.000<br>0.500<br>0.500
0.05	C	$a = 3.953$Å<br>$b = 3.953$Å<br>$c = 3.953$Å<br>$\alpha = 90°$<br>$\beta = 90°$<br>$\gamma = 90°$	Ba1<br>Ti1<br>O1	1a<br>1b<br>3c	0.000<br>0.500<br>0.000	0.000<br>0.500<br>0.500	0.000<br>0.500<br>0.500
0.06	C	$a = 3.956$Å<br>$b = 3.956$Å<br>$c = 3.956$Å<br>$\alpha = 90°$<br>$\beta = 90°$<br>$\gamma = 90°$	Ba1<br>Ti1<br>O1	1a<br>1b<br>3c	0.000<br>0.500<br>0.000	0.000<br>0.500<br>0.500	0.000<br>0.500<br>0.500
0.07	C	$a = 3.959$Å<br>$b = 3.959$Å<br>$c = 3.959$Å<br>$\alpha = 90°$<br>$\beta = 90°$<br>$\gamma = 90°$	Ba1<br>Ti1<br>O1	1a<br>1b<br>3c	0.000<br>0.500<br>0.000	0.000<br>0.500<br>0.500	0.000<br>0.500<br>0.500
0.08	C	$a = 3.962$Å<br>$b = 3.962$Å<br>$c = 3.962$Å<br>$\alpha = 90°$<br>$\beta = 90°$<br>$\gamma = 90°$	Ba1<br>Ti1<br>O1	1a<br>1b<br>3c	0.000<br>0.500<br>0.000	0.000<br>0.500<br>0.500	0.000<br>0.500<br>0.500

0.09	C	$a = 3.965$Å<br>$b = 3.965$Å<br>$c = 3.965$Å<br>$\alpha = 90°$<br>$\beta = 90°$<br>$\gamma = 90°$	Ba1<br>Ti1<br>O1	1a<br>1b<br>3c	0.000<br>0.500<br>0.000	0.000<br>0.500<br>0.500	0.000<br>0.500<br>0.500
0.10	C	$a = 3.968$Å<br>$b = 3.968$Å<br>$c = 3.968$Å<br>$\alpha = 90°$<br>$\beta = 90°$<br>$\gamma = 90°$	Ba1<br>Ti1<br>O1	1a<br>1b<br>3c	0.000<br>0.500<br>0.000	0.000<br>0.500<br>0.500	0.000<br>0.500<br>0.500
0.11	C	$a = 3.972$Å<br>$b = 3.972$Å<br>$c = 3.972$Å<br>$\alpha = 90°$<br>$\beta = 90°$<br>$\gamma = 90°$	Ba1<br>Ti1<br>O1	1a<br>1b<br>3c	0.000<br>0.500<br>0.000	0.000<br>0.500<br>0.500	0.000<br>0.500<br>0.500
0.12	C	$a = 3.975$Å<br>$b = 3.975$Å<br>$c = 3.975$Å<br>$\alpha = 90°$<br>$\beta = 90°$<br>$\gamma = 90°$	Ba1<br>Ti1<br>O1	1a<br>1b<br>3c	0.000<br>0.500<br>0.000	0.000<br>0.500<br>0.500	0.000<br>0.500<br>0.500
0.13	C	$a = 3.978$Å<br>$b = 3.978$Å<br>$c = 3.978$Å<br>$\alpha = 90°$<br>$\beta = 90°$<br>$\gamma = 90°$	Ba1<br>Ti1<br>O1	1a<br>1b<br>3c	0.000<br>0.500<br>0.000	0.000<br>0.500<br>0.500	0.000<br>0.500<br>0.500

0.14	C	$a = 3.981$Å<br>$b = 3.981$Å<br>$c = 3.981$Å<br>$\alpha = 90^\circ$<br>$\beta = 90^\circ$<br>$\gamma = 90^\circ$	Ba1<br>Ti1<br>O1	1a<br>1b<br>3c	0.000<br>0.500<br>0.000	0.000<br>0.500<br>0.500	0.000<br>0.500<br>0.500

Supplementary Note 8. La-DOPED $BaTiO_3$ SUPERCELLS

In this section, we perform supercell calculations to explicitly include real La atoms in $BaTiO_3$. We address two questions: 1) in the presence of real La atoms, are the conduction electrons uniformly distributed in doped $BaTiO_3$ and 2) in the presence of real La atoms, which atomic orbitals do the conduction electrons occupy?

To answer the first question: we study four representative supercells: 1) a $2 \times 2 \times 2$ supercell in which one Ba atom is replaced by one La atom to simulate a carrier density of $0.125e$/f.u., 2) a $3 \times 3 \times 3$ supercell in which one Ba atom is replaced by one La atom to simulate a carrier density of $0.037e$/f.u., 3) and 4) two different $3 \times 3 \times 3$ supercells in which two Ba atoms are replaced by two La atoms to simulate a carrier density of $0.074e$/f.u. We find that in the presence of real La atoms, the conduction electrons on Ti atoms are almost uniformly distributed as Supplementary Fig. 6 shows. In the three $3 \times 3 \times 3$ cases, the number of conduction electrons on Ti atoms has a small variation of about $0.003e$/Ti; and in the $2 \times 2 \times 2$ case the variation is negligible ($< 10^{-7}e$/Ti).

To answer the second question, we study two representative cases: a $2 \times 2 \times 2$ supercell with one Ba atom replaced by one La atom, shown in Supplementary Fig. 6**a** and a $3 \times 3 \times 3$ supercell with two Ba atoms replaced by two La atoms, shown in Supplementary Fig. 7**b**. In both cases, we show the iso-value surface of conduction electron density on each Ti atom (Supplementary Fig. 7**b** and **e**). The 'dice-like' shape of the isovalue surface indicates that the conduction electrons occupy Ti d_{xy}, d_{xz} and d_{yz} orbitals. Supplementary Fig. 7**c** and **f** show the density of states of the $2 \times 2 \times 2$ and $3 \times 3 \times 3$ supercells. The blue and green correspond to the total and Ti-d projected density of states. We find that there is a finite density of states around the Fermi level and it is mainly composed of Ti-d states. This reinforces that the doped electrons in $BaTiO_3$ are itinerant and occupy Ti-d orbitals.

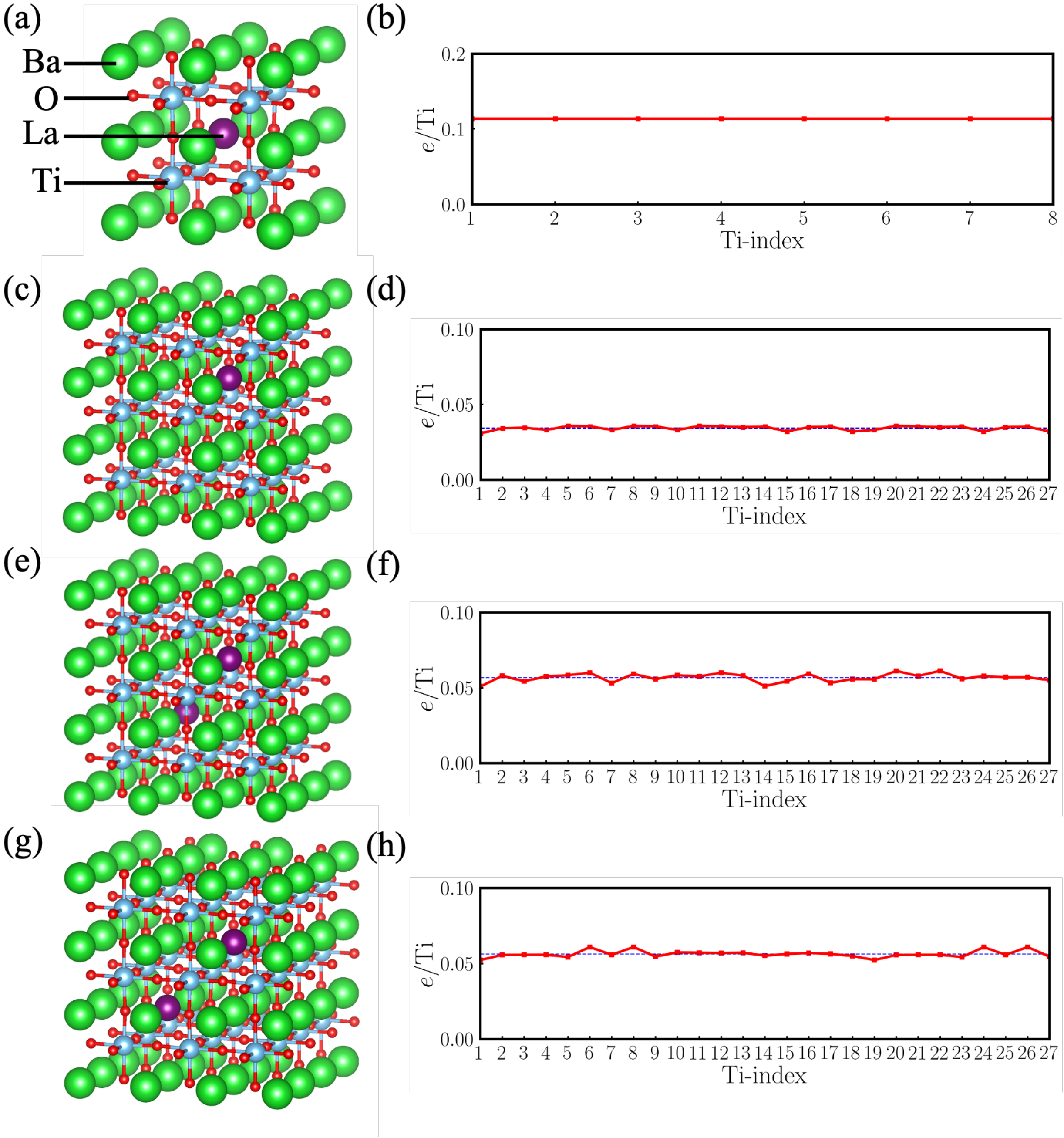

Supplementary Fig. 6. **a**) A $2 \times 2 \times 2$ supercell of La-doped $BaTiO_3$ (with one La atom in the supercell). **b**) The number of conduction electrons on each Ti atom in the $2 \times 2 \times 2$ supercell. **c**) A $3 \times 3 \times 3$ supercell of La-doped $BaTiO_3$ (with one La atom in the supercell). **d**) The number of conduction electrons on each Ti atom in the supercell. **e**) and **g**) Two $3 \times 3 \times 3$ supercells of La-doped $BaTiO_3$ in different configurations (with two La atoms in the supercell). **f**) and **h**) The number of conduction electrons on each Ti atom in the supercells.

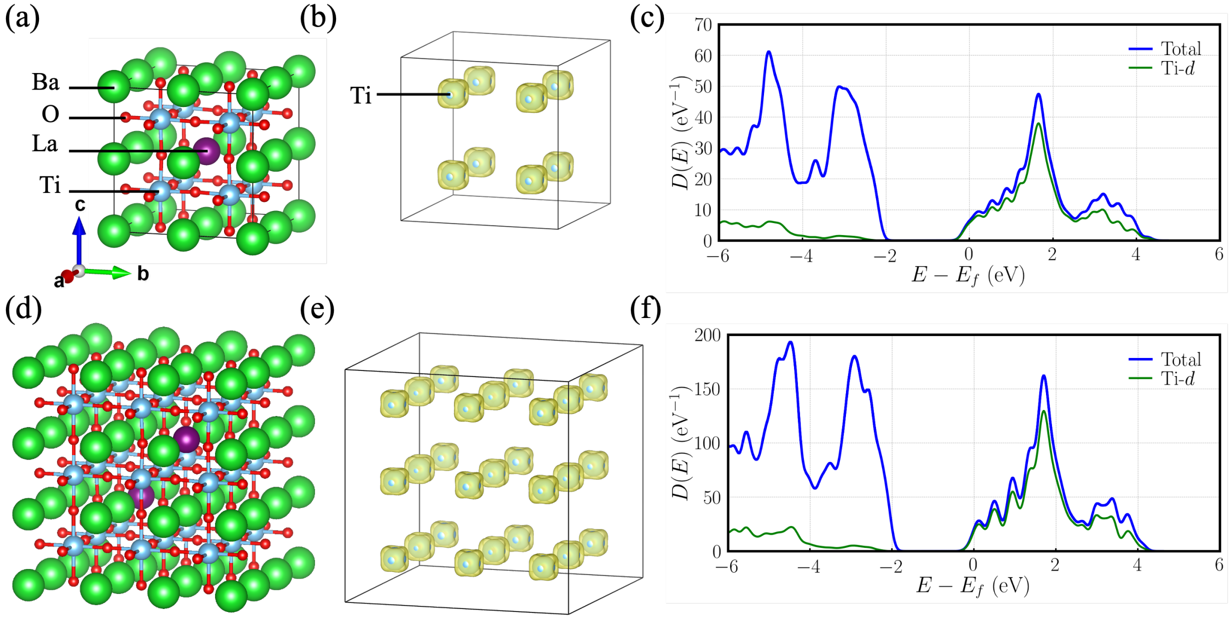

Supplementary Fig. 7. **a**) The crystal structure of $2 \times 2 \times 2$ La-doped $BaTiO_3$ supercell with one Ba atom replaced by one La atom. **b**) An isovalue surface of conduction electrons on Ti atom in the 222 La-doped $BaTiO_3$ supercell (for clarity only Ti atoms are shown). **c**) Total (blue) and Ti d-projected (green) density of states near the Fermi level of $2 \times 2 \times 2$ La-doped $BaTiO_3$ supercell. The Fermi level is shifted at zero point. **d**) The crystal structure of $3 \times 3 \times 3$ La-doped $BaTiO_3$ supercell with two Ti atoms replaced by two La atoms. **e**) An isovalue surface of conduction electrons on Ti atom in the $3 \times 3 \times 3$ La-doped $BaTiO_3$ supercell (for clarity only Ti atoms are shown). **f**) Total (blue) and Ti d-projected (green) density of states near the Fermi level of $3 \times 3 \times 3$ La-doped $BaTiO_3$ supercell. The Fermi level is shifted at zero point.

Supplementary Note 9. POSSIBILITY OF LONG-RANGE MAGNETIC ORDERING IN DOPED $BaTiO_3$

To study whether there might exist long-range magnetic ordering in doped $BaTiO_3$, we intentionally break spin symmetry and perform local spin density approximation (LSDA) calculations. In both $2\times2\times2$ and $3\times3\times3$ supercell calculations of $La_xBa_{1-x}TiO_3$, we do not find any magnetization in our LSDA calculations. Experimentally no long-range magnetic order is observed in La doped $BaTiO_3$ [10, 11]. We note that if we manually increase the correlation strength on Ti d orbitals, we can artificially stabilize magnetization in doped $BaTiO_3$ from LSDA+U calculations. This is due to Stoner instability [12]. Supplementary Fig. 8**a** shows such a calculation of $2\times2\times2$ supercell of $La_xBa_{1-x}TiO_3$ ($x = 0.125$), in which magnetization emerges when U_{Ti} is larger than 2 eV. However, it is well-known that $BaTiO_3$ is a band insulator with weak correlation effects [13]. More importantly, if we artificially increase the correlation strength on Ti d orbitals, we find that the polar distortions are suppressed in bulk $BaTiO_3$ because U_{Ti} changes the Ti-O hybridization [14]. Supplementary Fig. 8**b** shows the c/a ratio and Ti-O displacements δ of bulk $BaTiO_3$ as a function of U_{Ti}. As U_{Ti} is larger than 2 eV, the polar displacements in $BaTiO_3$ are completely suppressed, which is at odds with the experimentally observed ferroelectric property. Therefore based on our calculations and known experimental results, we conclude that long-range magnetic order (i.e. homogeneous magnetization) in doped $BaTiO_3$ is unlikely.

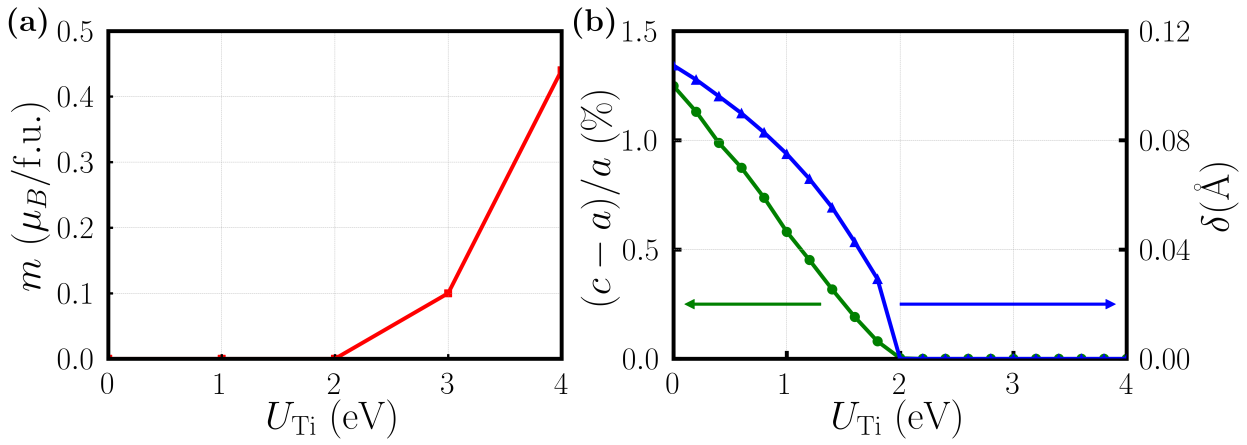

Supplementary Fig. 8. **a**) Total magnetic moment of a $2\times2\times2$ supercell $La_xBa_{1-x}TiO_3$ ($x = 0.125$) as a function of $U_{\rm Ti}$ from LSDA+U calculation. **b**) c/a ratio and Ti-O cation displacements δ of bulk $BaTiO_3$ as a function of $U_{\rm Ti}$ from LDA+U calculation.

Supplementary Note 10. METAL/$BaTiO_3$ INTERFACE

In this section, we study a Pt/$BaTiO_3$ heterostructure grown on a $SrTiO_3$ substrate. Pt is a representative electrode. We include 16 unit cells of $BaTiO_3$ and two Pt/TiO_2 interfaces. Supplementary Fig. 9**a** shows the optimized crystal structure. The two interfaces are not equivalent due to the presence of $BaTiO_3$ polarization. Supplementary Fig. 9**b** shows the layer-resolved Ti-O displacement δ in $BaTiO_3$ thin films. We find that at both interfaces, two or three atomic layers from either interface a bulk-like region emerges in which δ is almost uniform. Electrostatic doping can induce itinerant electrons into a region of the target material that is a few nanometers from the surface/interface [15]. Our calculations indicate that other than the very few atomic layers in the vicinity of surface/interface, $BaTiO_3$ thin films exhibit bulk-like structural properties with uniform cation displacements.

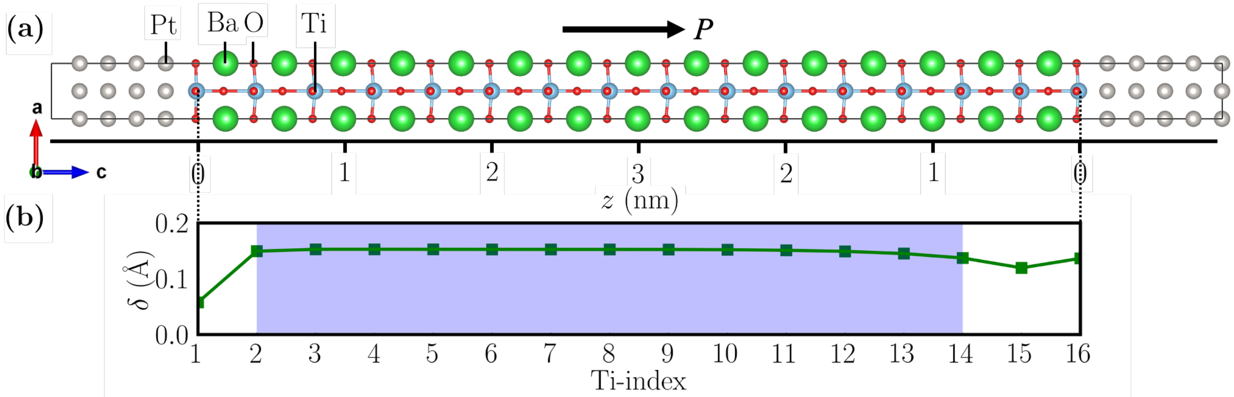

Supplementary Fig. 9. **a**) The crystal structure of Pt/$BaTiO_3$ heterostructure. The two Pt/TiO_2 interfaces are not symmetric due to the polarization in $BaTiO_3$ (the polarization points from left to right). The green, blue, red and gray balls are Ba, Ti, O and Pt atoms. **b**) The layer-resolved Ti-O displacement along the z-axis δ. The purple shade highlights the bulk-like region.

Supplementary Note 11. PHONON DISPERSION OF UNDOPED $SrTiO_3$

In this section, we show that density function theory and density functional perturbation theory are capable to describe the phonon spectrum of $SrTiO_3$.

Pristine $SrTiO_3$ is cubic at room temperature and develops an antiferrodistortive (AFD) rotation below 105 K (an out-of-phase oxygen octahedral rotation about the c-axis, $a^0a^0c^-$ in the Glazer notation) [16, 17]. Supplementary Fig. 10**a** shows the phonon band structure of cubic $SrTiO_3$ (space group $Pm\bar{3}m$, No. 221). Consistent with the previous study [18], imaginary phonon modes appear at M and R points. The imaginary phonon mode at R point corresponds to the AFD rotation ($a^0a^0c^-$ Glazer tilts). The unstable phonon mode at M point corresponds to an energetically less favorable $a^0a^0c^+$ Glazer tilts. Supplementary Fig. 10**b** shows the phonon band structure of the $SrTiO_3$ crystal structure with the AFD rotation (space group $I4/mcm$, No. 140), where the imaginary phonon modes disappear.

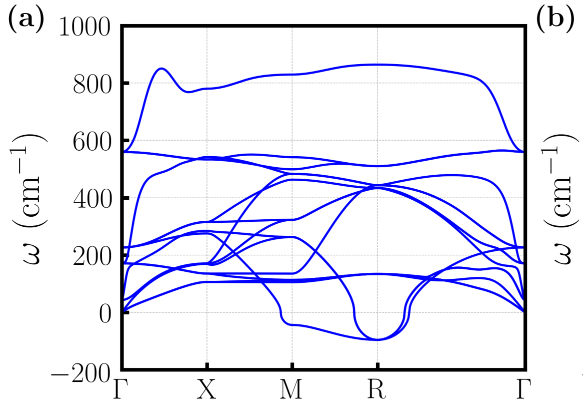

Supplementary Fig. 10. **a**) Phonon band structure of cubic $SrTiO_3$ (space group No. 221 $Pm\bar{3}m$). A **k**-point path including the high-symmetry points Γ(0,0,0)-X(0.5,0,0)-M(0.5,0.5,0)-R(0.5,0.5,0.5)-G(0,0,0) is used in the calculation. **b**) Phonon band structure of tetragonal $SrTiO_3$ with an out-of-phase oxygen octahedral rotation (space group No. 140 $I4/mcm$). A **k**-point path including the high-symmetry points Γ(0,0,0)-X(0,0,0.5)-Y(-0.246,0.246,0.5)-Σ(-0.373,0.373,0.373)-Γ(0,0,0)-Z(0.5,0.5,-0.5)-Y′(0.5,0.5,-0.246)-Σ′(0.373,0.627,-0.373)-N(0,0.5,0)-P(0.25,0.25,0.25)-Γ(0,0,0) is used in the calculation.

Supplementary Note 12. METAL-OXYGEN HYBRIDIZATION IN DOPED $BaTiO_3$

In this section, we study how the doped electrons may affect the metal-oxygen hybridization in $BaTiO_3$. Previous calculations show that p-d hybridization in transition metal oxides can be characterized by the occupancy of metal-d states [19, 20]. Therefore for doped $BaTiO_3$, we calculate the occupancy of Ti-d states as a function of electron doping. To provide more insights, we calculate the total occupancy of Ti-d states N_d^{tot}, the occupancy of Ti-d states in the valence bands N_d^{val} and the occupancy of Ti-d states in the conduction bands N_d^{cond}:

$$N_d^{\text{tot}} = \int_{-\infty}^{E_F} D(E)dE \tag{13}$$

$$N_d^{\text{val}} = \int_{-\infty}^{\text{in-gap}} D(E)dE \tag{14}$$

$$N_d^{\text{cond}} = \int_{\text{in-gap}}^{E_F} D(E)dE \tag{15}$$

where $D(E)$ is the density of states projected onto Ti-d orbitals. These three integrals are illustrated in Supplementary Fig. 11**a** and their values are shown in Supplementary Fig. 11**b**. We find that while N_d^{cond} almost linearly increases with electron doping, the total Ti-d occupancy N_d^{tot} changes much more slowly. This phenomenon is called "rehybridization effect" [21, 22]. As N_d^{cond} increases with electron doping, N_d^{val} decreases with electron doping because Ti-d and O-p states "rehybridize" in the valence states. As a result, N_d^{tot} changes slowly with electron doping. This indicates that electron doping does not significantly change the p-d hybridization in $BaTiO_3$.

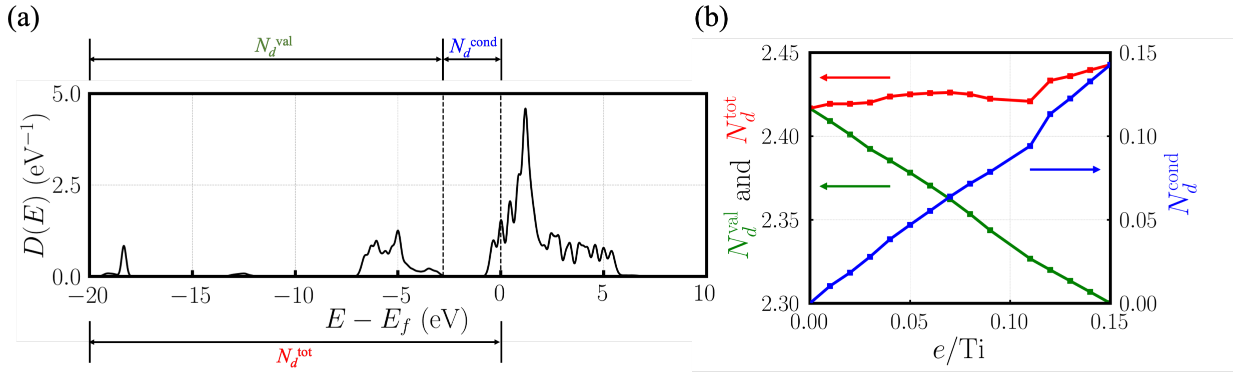

Supplementary Fig. 11. **a**) Illustration of the integral of Ti-d projected density of state for N_d^{val}, N_d^{cond} and N_d^{tot} (their definitions can be found in Eq. 13, 14 and 15). **b**) N_d^{val}, N_d^{cond} and N_d^{tot} for Ti-d states as a function of electron doping.

Supplementary Note 13. POLAR PHONONS AND PHONON DENSITY OF STATES OF $BaTiO_3$ AT DIFFERENT ELECTRON DOPING AND WITH DIFFERENT CRYSTAL SYMMETRIES

In this section, we study the phonon behavior of $BaTiO_3$ with electron doping. We select three representative dopings: 0, $0.07e$/f.u. and $0.14e$/f.u. In the un-doped case, the ground state structure is rhombohedral (R); at $0.07e$/f.u. doping, the ground state structure is tetragonal (T) and at $0.14e$/f.u. doping, the ground state structure is cubic (C). Supplementary Fig. 12 shows the polar phonons and phonon density of states of the ground state structure at different electron dopings. Supplementary Fig. 12**a** shows the polar phonon of the rhombohedral structure. The vibration mode of one polar phonon points along the [111] direction. The vibration mode of the other two degenerate polar phonons points perpendicular to the [111] direction. Supplementary Fig. 12**b** shows the polar phonon of the tetragonal structure. The vibration mode of one polar phonon points along the [001] direction. The vibration mode of the other two degenerate polar phonons points to the [100] and [010] directions. Supplementary Fig. 12**c** shows the polar phonon of the cubic structure. The vibration mode of the three degenerate polar phonons points along the [001],[010] and [100] directions. Supplementary Fig. 12**d**, **e** and **f** show the total and atomic-projected phonon density of states of the rhombohedral structure (undoped), of the tetragonal structure (at $0.07e$/f.u. doping) and of the cubic structure (at $0.14e$/f.u. doping). We find that since electron doping changes the ground state crystal structure of $BaTiO_3$, it affects the total phonon density of states. In particular, with electron doping, the frequency of the highest optical phonon modes decreases while the low-frequency peak (around 100 cm^{-1}) increases its height.

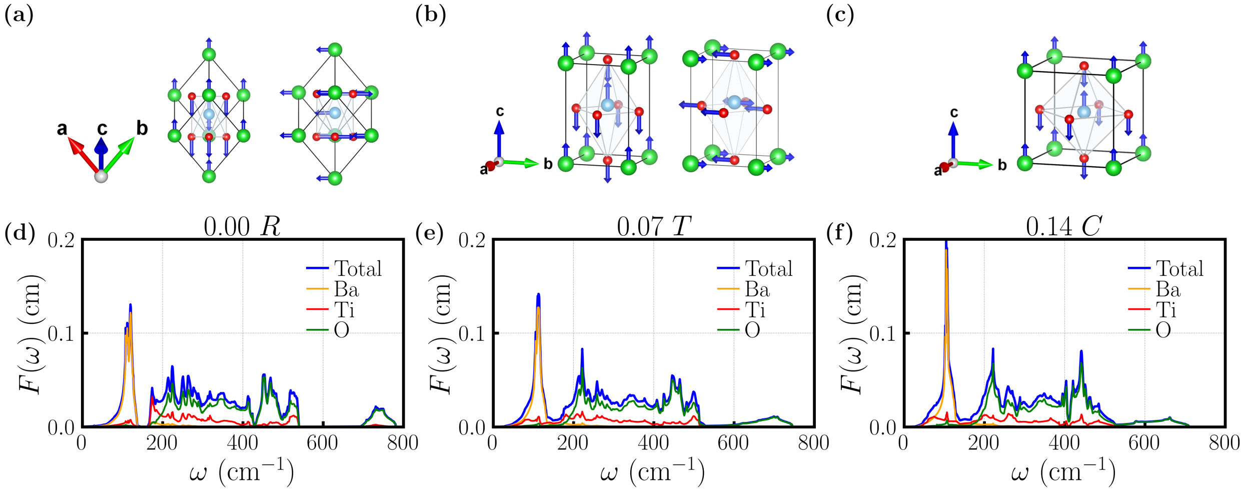

Supplementary Fig. 12. **a**) The vibration mode of the polar phonons in the rhombohedral $BaTiO_3$ (without doping). The vibration mode of one polar phonon points along the [111] direction and the vibration mode of the other two degenerate polar phonons points perpendicular to the [111] direction. **b**) The vibration mode of the polar phonons in the tetragonal $BaTiO_3$ (at $0.07e$/f.u. doping). The vibration mode of one polar phonon points along the [001] direction and the vibration mode of the other two degenerate polar phonons points along [100] and [010] directions. **c**) The vibration mode of the three degenerate polar phonons in the cubic $BaTiO_3$ (at $0.14e$/f.u. doping) points along [100], [010] and [001] directions. **d**) Phonon density of state of the rhombohedral $BaTiO_3$ (without doping). The blue, yellow, red and green correspond to the total, Ba-projected, Ti-projected and O-projected phonon densities of states. **e**) Phonon density of state of the tetragonal $BaTiO_3$ (at $0.07e$/f.u. doping). **f**) Phonon density of state of the cubic $BaTiO_3$ (at $0.14e$/f.u. doping).

Supplementary Note 14. TEST ON THE CHANGE OF DENSITY OF STATES WITH POLAR PHONON MODE

In this section, we study how the density of states at the Fermi level changes with polar phonon modes in doped $BaTiO_3$. We study tetragonal $BaTiO_3$ at $0.09e$/f.u. concentration as a representative example. Supplementary Fig. 13**a**) shows the schematic vibration mode of a polar phonon along the z-axis. Then we impose this polar phonon mode on the crystal structure with different phonon amplitudes. In Supplementary Fig. 13**b**), we compare the density of states of doped $BaTiO_3$ with and without the polar phonon mode imposed (denoted by $D^A(E)$ and $D^0(E)$, respectively). Here the amplitude $A = 0.12$Å. The inset of Supplementary Fig. 13 clearly shows that the density of states at the Fermi level can be strongly modulated by the polar phonon. Supplementary Fig. 13 shows the relative change of the density of states at the Fermi level $\left|\frac{D^A(E_f)-D^0(E_f)}{D^0(E_f)}\right|$ as a function of phonon amplitude A. We find a substantial change in $D^A(E_f)$ as the phonon amplitude A increases. This indicates that the polar phonon is strongly coupled to itinerant electrons in doped $BaTiO_3$.

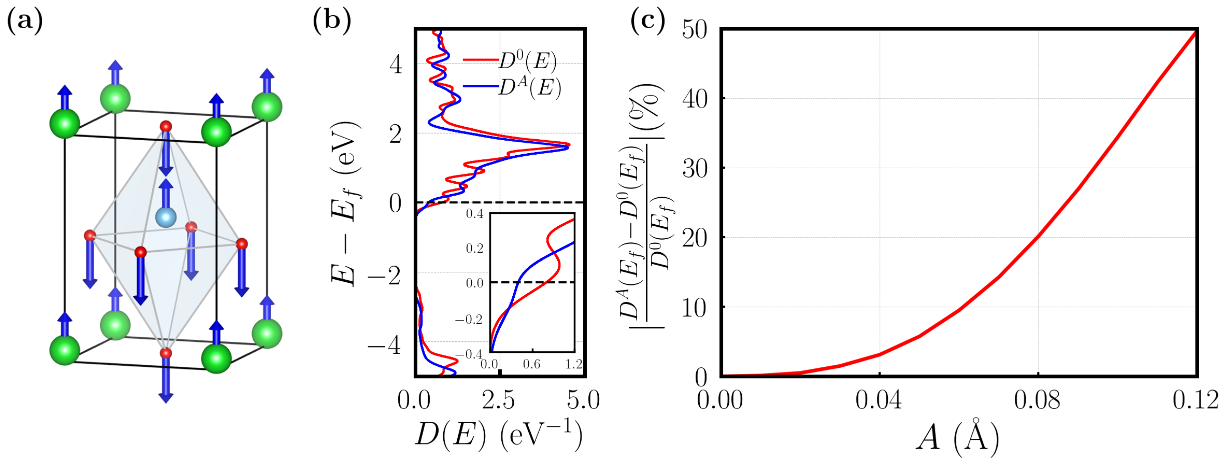

Supplementary Fig. 13. Doped $BaTiO_3$ at an electron concentration of 0.09e/f.u. **a**) The schematic vibration mode of the zone-center polar optical phonon along the long c axis (z-axis) of the tetragonal structure at 0.09e/f.u. **b**) Comparison of density of states with and without polar phonon distortions. The red curve corresponds to the fully relaxed structure and the blue curve corresponds to the structure with the polar phonon mode imposed (the amplitude of the phonon mode is 0.12 Å). The inset shows the near-Fermi-level density of states. **c**) The relative change of the density of state at the Fermi level $\left|\frac{D^A(E_f)-D^0(E_f)}{D^0(E_f)}\right|$ as a function of the polar phonon mode amplitude A.

Supplementary Note 15. ELECTRON-PHONON COUPLING OF DOPED $KTaO_3$

In this section we study the electron-phonon coupling of $KTaO_3$ at a representative electron concentration of $0.14e$/f.u. (based on the experiment [15]). Supplementary Fig. 14**a** compares the electron-phonon spectral function $\alpha^2F(\omega)$ between doped $KTaO_3$ (at $0.14e$/f.u.) and doped $BaTiO_3$ (at $0.09e$/f.u.). We find that while the electron-phonon coupling of doped $BaTiO_3$ at $0.09e$/f.u. is 0.61, doped $KTaO_3$ at $0.14e$/f.u. has a smaller electron-phonon coupling of 0.36. Supplementary Fig. 14**b** compares the superconducting transition temperature that is estimated by the Eliashberg equation. We find that since the electron-phonon coupling of $KTaO_3$ at $0.14e$/f.u. doping is smaller than that of $BaTiO_3$ at $0.09e$/f.u. doping, the $KTaO_3$ superconducting transition temperature is also smaller than the doped $BaTiO_3$ given that the two materials have similar Morel-Anderson pseudopotential μ^*.

It is very difficult (almost impossible) to use Eliashberg equation to determine a very small superconducting transition temperature (such as 50 mK for $KTaO_3$ at $0.14e$/f.u. doping). Our calculations of doped $KTaO_3$ find that if $\mu^*_{ij} \geq 0.1$, the superconducting transition temperature of $KTaO_3$ (at $0.14e$/f.u. doping) is no higher than 0.1 K, which is in reasonable agreement with the experiment [15].

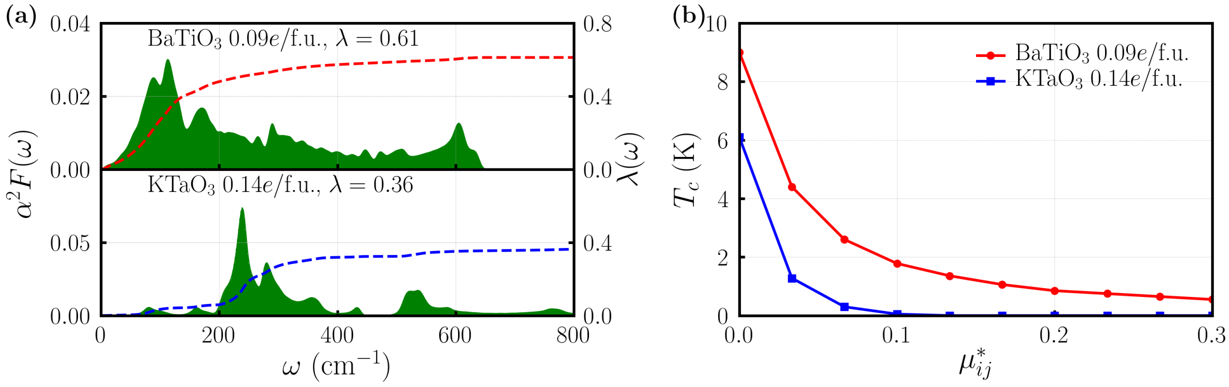

Supplementary Fig. 14. **a**) The electron-phonon spectral function $\alpha^2F(\omega)$ of $BaTiO_3$ at $0.09e$/f.u. doping (top) and of $KTaO_3$ at $0.14e$/f.u. doping (bottom). The dashed lines are the accumulative electron-phonon coupling. **b**) The superconducting transition temperature T_c of $BaTiO_3$ at $0.09e$/f.u. doping (red) and of $KTaO_3$ at $0.14e$/f.u. doping (blue) that is estimated by Eliashberg equation as a function of Morel-Anderson pseudopotential μ^*_{ij}.

Supplementary Note 16. ACOUSTIC PHONON OF DOPED $BaTiO_3$ AT $\mathbf{q}=X$

In this section, we study a specific phonon of $BaTiO_3$: the acoustic phonon at $\mathbf{q} = X$. Supplementary Fig. 15 compares the vibrational mode of $\mathbf{q} = X$ acoustic phonon of $BaTiO_3$ at $0.09e$/f.u. doping in the tetragonal structure (**a**), at $0.11e$/f.u. doping in the cubic structure (**b**) and at $0.11e$/f.u. doping under (001) 0.8% compressive strain (**c**). We find that in the cubic structure (**b**), Ba atoms are strictly frozen in this acoustic phonon, while in the tetragonal structure (**a** and **c**), Ba atoms also participate in the phonon mode. Correspondingly, the imaginary part of electron-phonon self-energy $\mathrm{Im}\Sigma_{\mathbf{q}\nu}$ of doped $BaTiO_3$ is very small in the cubic structure, but becomes sizable in the tetragonal structure. Due to the low frequency of acoustic phonons, the electron-phonon coupling $\lambda_{\mathbf{q}\nu}$ is substantially larger in the tetragonal structure than in the cubic structure.

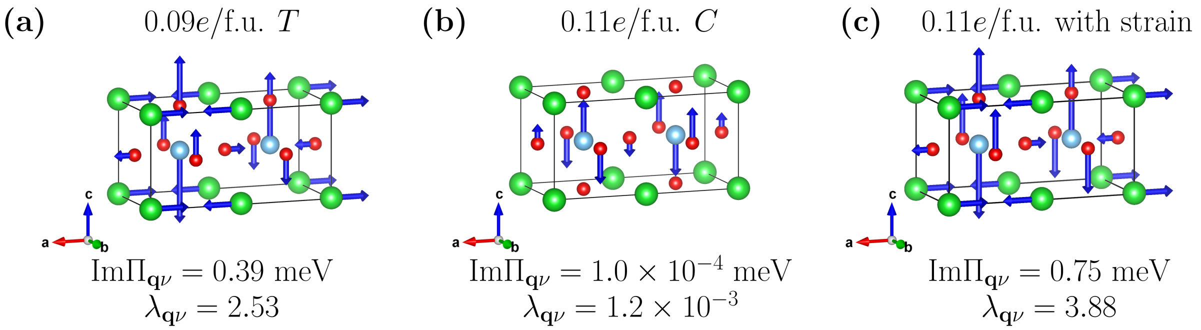

Supplementary Fig. 15. The vibrational mode of the acoustic phonon of doped $BaTiO_3$ at $\mathbf{q} = X$. **a**) at 0.09e/f.u. doping in the tetragonal structure. The imaginary part of electron-phonon self-energy Im$\Sigma_{\mathbf{q}\nu} = 0.39$ meV and the electron-phonon coupling $\lambda_{\mathbf{q}\nu} = 2.53$. **b**) at 0.11$e$/f.u. doping in the cubic structure. Im$\Sigma_{\mathbf{q}\nu} = 1.0 \times 10^{-4}$ meV and $\lambda_{\mathbf{q}\nu} = 1.2 \times 10^{-3}$. **c**) at 0.11$e$/f.u. doping under 0.8% compressive strain. Im$\Sigma_{\mathbf{q}\nu} = 0.75$ meV and $\lambda_{\mathbf{q}\nu} = 3.88$.

[1] Kwei, G. H., Lawson, A. C., Billinge, S. J. L. & Cheong, S. W. Structures of the ferroelectric phases of barium titanate. *The Journal of Physical Chemistry* **97**, 2368 –2377 (1993).

[2] Wieder, H. H. Electrical behavior of barium titanatge single crystals at low temperatures. *Phys. Rev.* **99**, 1161–1165 (1955).

[3] Margine, E. R. & Giustino, F. Anisotropic migdal-eliashberg theory using wannier functions. *Phys. Rev. B* **87**, 024505 (2013).

[4] Giustino, F. Electron-phonon interactions from first principles. *Rev. Mod. Phys.* **89**, 015003 (2017).

[5] Ponce, S., Margine, E., Verdi, C. & Giustino, F. EPW: Electronphonon coupling, transport and superconducting properties using maximally localized Wannier functions. *Computer Physics Communications* **209**, 116–133 (2016).

[6] Noffsinger, J. *et al.* EPW: A program for calculating the electronphonon coupling using maximally localized wannier functions. *Computer Physics Communications* **181**, 2140–2148 (2010).

[7] Chen, J., Millis, A. J. & Reichman, D. R. Intermolecular coupling and superconductivity in $PbMo_6S_8$ and other chevrel phase compounds. *Phys. Rev. Materials* **2**, 114801 (2018).

[8] Allen, P. B. & Dynes, R. C. Transition temperature of strong-coupled superconductors reanalyzed. *Phys. Rev. B* **12**, 905–922 (1975).

[9] Migdal, A. Interaction between electrons and lattice vibrations in a normal metal. *Soviet Physics Journal of Experimental and Theoretical Physics* **7**, 996–1001 (1958).

[10] Takahashi, K. S. *et al.* Polar metal phase stabilized in strained La-doped $BaTiO_3$ films. *Scientific Reports* **7** (2017).

[11] Zhang, A. *et al.* Conductivity, charge transport, and ferroelectricity of La-doped $BaTiO_3$ epitaxial thin films. *Journal of Physics D: Applied Physics* **53**, 025301 (2019).

[12] Stoner, E. C. Collective electron ferronmagnetism. *Proceedings of the Royal Society of London. Series A. Mathematical and Physical Sciences* **165**, 372–414 (1938).

[13] Kolodiazhnyi, T., Tachibana, M., Kawaji, H., Hwang, J. & Takayama-Muromachi, E. Persistence of ferroelectricity in $BaTiO_3$ through the insulator-metal transition. *Phys. Rev. Lett.* **104**, 147602 (2010).

[14] Benedek, N. A. & Birol, T. ferroelectric metals reexamined: fundamental mechanisms and design considerations for new materials. *J. Mater. Chem. C* **4**, 4000–4015 (2016).

[15] Ueno, K. *et al.* Discovery of superconductivity in $KTaO_3$ by electrostatic carrier doping. *Nature Nanotechnology* **6**, 408–412 (2011).

[16] Heifets, E., Kotomin, E. & Trepakov, V. A. Calculations for antiferrodistortive phase of $SrTiO_3$ perovskite: hybrid density functional study. *Journal of Physics: Condensed Matter* **18**, 4845–4851 (2006).

[17] Rimai, L. & deMars, G. A. Electron paramagnetic resonance of trivalent gadolinium ions in strontium and barium titanates. *Phys. Rev.* **127**, 702–710 (1962).

[18] Aschauer, U. & Spaldin, N. A. Competition and cooperation between antiferrodistortive and ferroelectric instabilities in the model perovskite $SrTiO_3$. *Journal of Physics: Condensed Matter* **26**, 122203 (2014).

[19] Wang, X. *et al.* Covalency, double-counting, and the metal-insulator phase diagram in transition metal oxides. *Phys. Rev. B* **86**, 195136 (2012).

[20] Park, H., Millis, A. J. & Marianetti, C. A. Computing total energies in complex materials using charge self-consistent DFT + DMFT. *Phys. Rev. B* **90**, 235103 (2014).

[21] Marianetti, C. A., Kotliar, G. & Ceder, G. Role of hybridization in Na_xCoO_2 and the effect of hydration. *Phys. Rev. Lett.* **92**, 196405 (2004).

[22] Grisolia, M. N. *et al.* Hybridization-controlled charge transfer and induced magnetism at correlated oxide interfaces. *Nature Physics* **12**, 484–492 (2016).
